# Quantitative neuroimaging measures of myelin in the healthy brain and in multiple sclerosis

**DOI:** 10.1002/hbm.24510

**Published:** 2019-01-15

**Authors:** Jonathan O'Muircheartaigh, Irene Vavasour, Emil Ljungberg, David K. B. Li, Alexander Rauscher, Victoria Levesque, Hideki Garren, David Clayton, Roger Tam, Anthony Traboulsee, Shannon Kolind

**Affiliations:** ^1^ Department of Forensic and Neurodevelopmental Sciences Institute of Psychiatry Psychology and Neuroscience, King's College London London United Kingdom; ^2^ Centre for the Developing Brain, Department of Perinatal Imaging and Health St. Thomas' Hospital, King's College London London United Kingdom; ^3^ Department of Neuroimaging Institute of Psychiatry Psychology and Neuroscience, King's College London London United Kingdom; ^4^ MRC Centre for Neurodevelopmental Disorders King's College London London United Kingdom; ^5^ Department of Radiology University of British Columbia Vancouver British Columbia Canada; ^6^ MS/MRI Research Group, Djavad Mowafaghian Centre for Brain Health University of British Columbia Vancouver British Columbia Canada; ^7^ Department of Physics and Astronomy University of British Columbia Vancouver British Columbia Canada; ^8^ Department of Pediatrics University of British Columbia Vancouver British Columbia Canada; ^9^ Genentech, Inc. South San Francisco California; ^10^ School of Biomedical Engineering University of British Columbia Vancouver British Columbia Canada; ^11^ Division of Neurology, Department of Medicine University of British Columbia Vancouver British Columbia Canada

**Keywords:** individual differences, magnetization transfer ratio, multiple sclerosis, myelin, myelin water imaging, quantitative MRI, relaxometry

## Abstract

Quantitative magnetic resonance imaging (MRI) techniques have been developed as imaging biomarkers, aiming to improve the specificity of MRI to underlying pathology compared to conventional weighted MRI. For assessing the integrity of white matter (WM), myelin, in particular, several techniques have been proposed and investigated individually. However, comparisons between these methods are lacking. In this study, we compared four established myelin‐sensitive MRI techniques in 56 patients with relapsing–remitting multiple sclerosis (MS) and 38 healthy controls. We used T2‐relaxation with combined GRadient And Spin Echoes (GRASE) to measure myelin water fraction (MWF‐G), multi‐component driven equilibrium single pulse observation of T_1_ and T_2_ (mcDESPOT) to measure MWF‐D, magnetization‐transfer imaging to measure magnetization‐transfer ratio (MTR), and T_1_ relaxation to measure quantitative T_1_ (qT_1_). Using voxelwise Spearman correlations, we tested the correspondence of methods throughout the brain. All four methods showed associations that varied across tissue types; the highest correlations were found between MWF‐D and qT_1_ (median ρ across tissue classes 0.8) and MWF‐G and MWF‐D (median ρ = 0.59). In eight WM tracts, all measures showed differences (*p* < 0.05) between MS normal‐appearing WM and healthy control WM, with qT1 showing the highest number of different regions (8), followed by MWF‐D and MTR (6), and MWF‐G (*n* = 4). Comparing the methods in terms of their statistical sensitivity to MS lesions in WM, MWF‐D demonstrated the best accuracy (*p* < 0.05, after multiple comparison correction). To aid future power analysis, we provide the average and standard deviation volumes of the four techniques, estimated from the healthy control sample.

## 1 INTRODUCTION

Quantitative magnetic resonance imaging (MRI) techniques that are used as markers of white matter (WM) and myelin content include magnetic resonance relaxation‐based and magnetization transfer‐based methods. Both approaches provide WM *contrast* related to myelin, but are predicated on different assumptions and models, and may not directly correspond to each other (Alexander et al., 2011; De Santis, Drakesmith, Bells, Assaf, & Jones, 2014). Diseases of WM, and multiple sclerosis (MS) in particular have motivated the search for quantitative and specific MRI measures of myelin content as measures of treatment response and remission (Filippi et al., 2012). Although conventional clinical MRI protocols for monitoring MS include qualitative T_1_ and T_2_ weighted images, these lack specificity to the underlying pathology and are difficult to compare across imaging sites and scanners (Barkhof, Calabresi, Miller, & Reingold, 2009). Quantitative MRI can supplement standard clinical imaging by providing detection of changes within normal appearing tissue, as well as biological characterization of these alterations (MacKay and Laule, 20162016).

Multicomponent relaxation‐based techniques model tissue water compartments by observing the difference in relaxation times. For example, water trapped between myelin bilayers and the water inside or outside of axons have different T_1_ and T_2_ relaxation times, and by measuring the relative signal contribution from each component, a measure such as the myelin water fraction (MWF) can be obtained (Deoni, Rutt, Arun, Pierpaoli, & Jones, 2008; MacKay et al., 2006). The MWF is positively correlated with myelin content, and therefore lower MWF is thought to reflect a decrease in myelin content (Laule et al., 2008). Quantification of the macromolecular content as a proxy for myelin can be performed using the magnetization transfer ratio (MTR; Filippi et al., 1995; Schmierer, Scaravilli, Altmann, Barker, & Miller, 2004), the more advanced quantitative magnetization transfer (qMT; Schmierer et al., 2007), or the macromolecular tissue volume (MTV; Mezer et al., 2013). A decrease in MTR is commonly interpreted as a decrease of myelin. T_1_‐relaxation time (commonly referred to as quantitative T_1_, qT_1_) is also found in the literature as a quantitative biomarker for pathology (Margaret Cheng, Stikov, Ghugre, & Wright, 2012), with increased qT_1_ associated with myelin loss.

Many of these techniques have been investigated in MS (Harrison et al., 2013; Kitzler et al., 2012; Kolind et al., 2012; Ontaneda, Thompson, Fox, & Cohen, 2016). For multi‐echo techniques, Laule et al. (2006, 2008) demonstrated strong associations across tissue types between histological staining of myelin and the T_2_‐relaxation‐based MWF obtained with a multi‐echo spin echo sequence. The acquisition can be achieved in clinically feasible times using a combined GRadient And Spin Echo (GRASE) sequence (Prasloski et al., 2012); the MWF measurement obtained using a GRASE is here termed MWF‐G. An alternative, and more recently, multicomponent relaxation technique is mcDESPOT, from which the fraction of signal attributed to myelin water (here called MWF‐D), similar to MWF‐G, can be obtained. To date, there is no human ex vivo histology validation of mcDESPOT. However, Hurley et al. (2010) used the shaking pup preclinical model of dysmyelination and showed that MWF‐D was sensitive, at the very least, to an absence of myelin. Wood et al. (2016) performed whole‐brain mcDESPOT in a cuprizone mouse model and found a decrease in MWF‐D, related to the expected demyelination. MTR has been validated against postmortem tissue using histopathology, showing good sensitivity to myelin and demyelination (Schmierer et al., 2004). However, since MTR measures the interaction between macromolecular protons and bulk water, it is also related to the total water content of tissue and therefore, in cases of WM injury, edema, and inflammation can obscure the myelin‐related signal (Gareau, Rutt, Karlik, & Mitchell, 2000; Vavasour, Laule, Li, Traboulsee, & MacKay, 2011). In addition, some postmortem studies have found an even larger association between MTR and axonal count (Mottershead et al., 2003; Van Waesberghe et al., 1999). Single component measures of qT_1_ have also been shown to correlate strongly with histological staining for myelin (Schmierer et al., 2007). However, interpretations of changes in T_1_ are confounded by the known relationship between 1/T_1_ and 1/water content (Fatouros, Marmarou, Kraft, Inao, & Schwarz, 1991; Gelman, Ewing, Gorell, Spickler, & Solomon, 2001; Kamman, Go, Brouwer, & Berendsen, 1988; Rooney et al., 2007). Thus, it is difficult to deduce whether changes in T_1_ are due to demyelination and/or changes in total water content.

The common feature of most of these validation studies is that they have shown that the quantitative image *contrast* is similar between myelin‐sensitive techniques, though they do not correspond only to the amount of myelin present. Even histological stains, such as luxol fast blue, provide a *contrast;* the optical density of these contrasts does not in itself provide a quantification of tissue content (Stüber et al., 2014). In general, strong correlations *across* tissue types (in the brain typically white and gray matter) indicate that two methods have similar tissue contrast, an important precondition. However, the level of consistency between quantities from the different methods is unclear.

In this study, we investigated the cross‐subject similarity between four prominent quantitative MRI techniques that are associated with WM content: Multi‐echo T_2_ relaxation, mcDESPOT, MTR, and qT_1_. The techniques were compared against each other to determine which technique best separates tissue between MS patients and healthy controls. Finally, we investigated the relative sensitivity of each of these techniques to lesional WM in individual patients with MS.

## 2 METHODS

### 2.1 Participants

The study cohort consisted of 56 patients with relapsing–remitting MS (19:37 male:female; mean age = 37 years, range = 20–55; median EDSS = 2.0, EDSS range = 0.0–4.0) recruited as part of a clinical trial of ocrelizumab versus interferon beta 1a (OPERA II; NCT01412333) (Hauser et al., 2017), and 38 age and gender‐matched healthy controls (13 male; mean age = 34, range = 20–53). As described in Hauser et al. (2017), to be eligible to take part in the study, MS patients were required to have at least two undocumented clinical relapses within the previous 2 years or one clinical relapse in the year before screening. MRI data needed to show MS consistent abnormalities and no neurological worsening over the 30 days prior to screening. In addition, participants were excluded if they had a disease duration of over 10 years.

Data presented here were acquired at baseline, prior to the initiation of treatment. Details of the patient and control cohort are presented in Table 1. Thirty‐two MS patients did not have an MTR scan due to time constraints, thus 24 of the 56 patients had all four MRI techniques acquired (MS Patients Subset in Table 1). This subset of 24 was used when comparing imaging methods in the MS group only. This study was approved by the University of British Columbia Clinical Research Ethics Board and all subjects provided written informed consent before participating in the study.

**Table 1:** Overview of the study cohort

	MS patients (*N* = 56)	MS patients subset* (*N* = 24)	Healthy controls (*N* = 38)
Age (years)	Mean: 37	Mean: 37	Mean: 35, range: 20–53
	Range 20–55	Range 20–53	
Sex	19 M, 37 F	9 M, 15 F	13 M, 25 F
EDSS	Median: 2.0	Median: 2.0	
	Range: 0–4.0	Range: 0–4.0	
Lesion volume (mm^3^)	Mean: 7394, median: 3523, range: 446–47,370	Mean: 4031, median: 2327, range: 214–14,047	
Number of lesions (count)	Mean: 26.5, median: 22.5, range: 3–71	Mean: 33, median: 30.5, range: 3–71	

* Only patients with all 4 quantitative imaging techniques collected.

### 2.2 MRI data acquisition

All MR imaging was performed on a Philips 3T Achieva scanner (Best, The Netherlands) using an eight‐channel head RF array coil. For localization, a true midline sagittal scan (TR = 1900 ms, TI = 800 ms, TE = 10 ms) and a quick T_2_‐weighted scan (TR = 2,792 ms, TE = 90 ms, 60 axial slices acquired at 3 mm slice thickness, in‐plane voxel size = 1 × 1 mm^2^, field of view [FOV] = 250 × 188 × 180 mm^3^) were performed. A 3D‐T1‐weighted gradient echo scan (TR = 28 ms, TE = 4 ms, 60 axial slices acquired at 3 mm slice thickness, in‐plane voxel size = 1 × 1 mm^2^, flip angle = 27°, FOV = 250 × 188 × 180 mm^3^) was also acquired. For lesion identification, a proton density weighted (PDw) (TR = 2000 ms, TE = 10 ms, 60 axial slices acquired at 3 mm slice thickness, in‐plane voxel size = 1 × 1 mm^2^, FOV = 250 × 200 × 180 mm^3^, echo train length [ETL] = 3), a T_2_‐weighted (TR = 6,100 ms, TE = 80 ms, 60 axial slices acquired at 3 mm slice thickness, in‐plane voxel size = 1 × 1 mm^2^, FOV = 250 × 188 × 180 mm^3^, ETL = 8), and a FLAIR (TR = 9,000 ms, TE = 80 ms, TI = 2,500 ms, 60 axial slices acquired at 3 mm slice thickness, in‐plane voxel size = 1 × 1 mm^2^, FOV = 250 × 188 × 180 mm^3^, ETL = 12) sequence were collected. Table 2 summarizes all pulse sequence parameters.

**Table 2:** MRI sequence pulse sequence parameters

							mcDESPOT			
	Quick T2	3DT1	PD lesion	T2 lesion	FLAIR	GRASE	SPGR	bSSFP	IRSPGR	MTR
Resolution acquired/reconstructed [mm2]	1 × 1.2/1 × 1	1 × 1/1 × 1	1 × 1/1 × 1	1 × 1/1 × 1	1 × 1	1 × 1	1.7 × 1.7	1.7 × 1.7	1.7 × 1.7	1 × 1
Slice thickness acquired/reconstructed [mm]	3/3	3/3	3/3	3/3	3/3	5/2.5	1.7/1.7	1.7/1.7	3.4/1.7	5
Number of reconstructed slices	60	60	60	60	60	40	92	92	92	20
Field of view (FOV)	250 × 188 × 180	250 × 188 × 180	250 × 200 × 180	250 × 188 × 180	250 × 188 × 180	230 × 192 × 100	220 × 160 × 220	220 × 160 × 220	220 × 160 × 220	230 × 192 × 100
Echo time (TE) [ms]	90	4	10	80	80	10	3.6	2.9	3.2	3.7
Repetition time (TR) [ms]	2,792	28	2000	6,100	9,000	1,000	6.5	5.8	6.5	85
Inversion time (TI) [ms]	n/a	n/a	n/a	n/a	2,500	n/a	n/a	n/a	450	n/a
Flip angle (α) [deg]	120^a^	27	n/a	n/a	120^a^	180^a^	2, 3, 4, 6, 9, 13, 18	7, 11, 15, 19, 24, 30, 47	5	18
Echo train length (ETL)	22	n/a	3	8	12	32	n/a	n/a	n/a	n/a
Scan mode	MS	3D	MS	MS	MS	3D	3D	3D	3D	3D
Parallel imaging (SENSE)	No	No	No	No	No	2 (RL)	2 (AP)	2 (AP)	2 (AP)	2 (RL)
Partial k‐space	No	No	No	No	No	No	0.6	0.6	No	0.6
Acquisition time [min]	2	5.3	4.5	4.9	7.2	14.5	3^b^	2.7^b^	1.1	3.5

MS, Multislice (2D); RL, right–left; AP, anterior–posterior; n/a, not applicable.

a Refocusing flip angle.

b Total acquisition time for all flip angles.

### 2.3 Quantitative imaging sequences

#### 2.3.1 GRASE

Multi‐echo T_2_ relaxation was measured using a GRASE sequence (TR = 1,000 ms, TE = 10 ms, ETL = 32, 20 axial slices acquired at 5 mm slice thickness and reconstructed to 40 slices at 2.5 mm slice thickness, slice oversampling factor = 1.3, in‐plane voxel size = 1 × 1 mm^2^, FOV = 230 × 192 × 100 mm^3^, EPI factor = 3) (Prasloski, Rauscher, et al., 2012).

#### 2.3.2 mcDESPOT

The mcDESPOT protocol was composed of a series of sagittally oriented spoiled gradient recalled echo (SPGR) and balanced steady‐state free procession (bSSFP) acquisitions across a range of flip angles (α) as well as an inversion‐recovery‐prepared SPGR (IR‐SPGR) scan for correction of flip angle inhomogeneity (Deoni, 2011). A common isotropic voxel size of 1.7 × 1.7 × 1.7 mm^3^ and field of view of 220 × 160 × 220 mm^3^ were used for all images. Scan parameters for the individual sequences were: SPGR: TR = 6.5 ms; TE = 3.6 ms; α = [2, 3, 4, 6, 9, 13, 18]°, bSSFP: TR = 5.8 ms; TE = 2.9 ms; α = [7, 11, 15, 19, 24, 30, 47]°, IRSPGR: TR = 6.5 ms; TE = 3.2 ms; α = 5°; TI = 450 ms; for the bSSFP volumes, all flip angles were acquired with phase‐cycling patterns of 0° and 180° for correction of off‐resonance effects (Deoni, 2011).

#### 2.3.3 Magnetization transfer imaging

The magnetization transfer imaging acquisition consisted of a 3D gradient echo sequence (TR = 85 ms, TE = 3.7 ms, 20 axial slices acquired at 5 mm slice thickness and reconstructed to 40 slices at 2.5 mm slice thickness, in‐plane voxel size = 1 × 1 mm^2^, FOV = 230 × 192 × 100 mm^3^, α = 18°, with and without an off‐resonance RF pulse centered 1.1 kHz below the water frequency, sinc‐gaussian envelope of duration = 15 ms, bandwidth = 190 Hz, amplitude = 2.3 × 10^−6^ T).

### 2.4 Quantitative image processing

The signal decay curve obtained by the GRASE T_2_ relaxation sequence was modeled by multiple exponential components and the T_2_ distribution was estimated using nonnegative least squares with the extended phase graph algorithm as well as spatial regularization (Prasloski, Mädler, Xiang, MacKay, & Jones, 2012; Whittall & MacKay, 1989; Yoo et al., 2015). The MWF‐G was calculated in each image voxel as the ratio of the area under the T_2_ distribution with 10 ≤ T_2_ ≤ 40 ms to the total area under the distribution. From the mcDESPOT data, the MWF‐D and qT_1_ maps were calculated using the processing methods outlined in Deoni and Kolind (2015); briefly, the SPGR and IR‐SPGR scans were used for DESPOT1 with High‐speed Incorporation of RF Field Inhomogeneities (DESPOT1‐HIFI) analysis (Deoni, 2007), resulting in maps of the global T1 and the B1 field. The bSSFP data (acquired with two phase‐cycling schemes) and the global T1 and B1 maps were then used to calculate global T2 and B0 field maps using DESPOT2 with full modeling (DESPOT2‐FM) analysis (Deoni, 2009). Finally, the B0 and B1 maps combined with the SPGR and bSSFP (with both phase‐cycling schemes) were used to calculate the MWF using stochastic region contraction (Deoni & Kolind, 2015). MTR maps were created by calculating (*M*_0_ − *M*_s_)/*M*_0_ × 100 for each voxel, where *M*_0_ is the image without the saturation pulse and *M*_s_ is the image with the saturation pulse.

### 2.5 Registration to template space

To provide a representative common space for the datasets collected here, a study‐specific template was constructed, from the high flip angle (18°) T_1_w SPGR image of 40 subjects. This was performed using the ANTs package (version 2.1.x; https://github.com/stnava/ANTs) and implemented using the *buildtemplateparallel.sh* script (Avants et al., 2011). We chose the high flip angle T_1_w image as it has good gray/WM tissue contrast and has isotropic resolution whereas the 3D gradient echo T_1_w image had nonisotropic voxels. This template was built from an age‐matched subgroup of the subjects with and without MS (40 images in total, 10 male and 10 female per group, with an average age of 36). A final nonlinear transformation was calculated from this template space to MNI space, also using the ANTs package.

**Figure 1:**
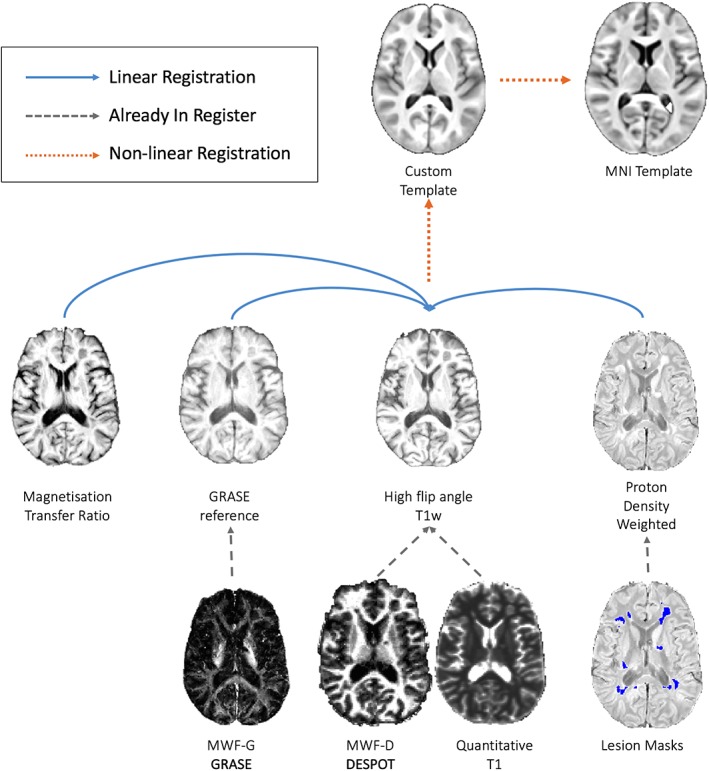
Registration pipeline for resampling all images to a common template [Color figure can be viewed at http://wileyonlinelibrary.com]

Within‐subject, MTR (image without the saturation pulse), MWF‐G (first echo GRASE image), and PDw images were registered to the high flip‐angle T_1_‐weighted SPGR images from the mcDESPOT sequence using rigid registration (FLIRT; Jenkinson & Smith, 2001). For each subject, this T_1_w image was then nonlinearly registered to the study‐specific template (ANTs; Avants, Epstein, Grossman, & Gee, 2008). Finally, using the combined registrations, the MWF‐G, MWF‐D, qT_1_, and MTR quantitative maps were registered and resampled into 1 mm isotropic MNI space in a single linear interpolation step. In addition, for the patient sample, the lesion masks (which are described below) were registered and resampled into MNI space using nearest neighbor interpolation. The registration pipeline is outlined in Figure 1.

### 2.6 Delineation of lesion and regions of interest

Lesions were identified on the T_2_w, PDw, and FLAIR for each subject by an experienced radiologist, placing one or more seed points where a lesion was identified. Segmentation was then performed automatically using the provided seed points according to the method described by Tam, Traboulsee, Riddehough, Sheikhzadeh, and Li (2011) and McAusland et al. (2010). In addition to the semi‐automatically defined lesions masks, additional masks of the boundary of each lesion (2 mm dilation of the semi‐automatically defined lesion) were created and are referred to here as peri‐lesional tissue.

Tissue masks were derived from the Harvard–Oxford atlas distributed as part of the FSL package (https://fsl.fmrib.ox.ac.uk/fsl). Three masks were created, one for gray matter, one for WM, and one encompassing subcortical structures (amygdala, hippocampus, basal ganglia, and thalamus; Figure 3). Anatomical regions of interest in WM were defined in standard space using the JHU probabilistic tractography atlas (Hua et al., 2008). These regions were then combined with the group WM tissue mask to ensure only WM was included. Eight major WM bundles were investigated: anterior thalamic radiation (ATR), cingulum, cortico‐spinal tract (CSP), forceps major, forceps minor, inferior fronto‐occipital fasciculus (IFO), inferior longitudinal fasciculus (ILF), and superior longitudinal fasciculus (SLF). For patients, the tissue and WM regions of interest excluded both the lesion and perilesional tissue, thus full brain WM segmentation is referred to as normal‐appearing WM (NAWM).

### 2.7 Summary and correlation maps

A group average quantitative map and a standard deviation (*SD*) map were created for each modality from the healthy control group. Spearman correlation maps between the different quantitative values at every voxel were calculated for the whole brain using 3dTcorrelate, part of the AFNI package (Cox, 1996). Correlation maps and histograms (per tissue class) of the correlation coefficients were produced.

### 2.8 Receiver operator characteristic maps

For all quantitative data sets available in the patient population, individual‐patient voxelwise difference maps were created by Z‐normalizing the patient's parameter maps to the mean and *SD* of the healthy control sample. In the 24 patients for whom all four quantitative sequences were successfully acquired (see Table 1 for characteristics), receiver operator characteristic (ROC) curves were constructed for each individual dataset, testing the accuracy of each MRI method for lesional WM detection (Fawcett, 2006). These curves were generated by using a range of Z‐score cutoffs between the minimum and maximum Z‐score and comparing whether each voxel was inside or outside of the Z‐score cutoff and whether the same voxel was included or not included within the semi‐automated lesion masks. For every threshold, the proportion of voxels outside of the Z‐score cutoff (considered to be lesional from the advanced MR technique) that were also inside the semi‐automated lesion mask is equal to the true positive rate. The percentage of voxels above the Z‐score cutoff but outside of the semi‐automated lesion mask is equal to the false positive rate. To compare the sensitivity/specificity of the four methods to manually defined lesions, the area under the curve (AUC) of ROCs from each dataset was calculated for each subject and compared pairwise across all methods using a nonparametric Friedman test. Post hoc inter‐method comparisons were tested using Wilcoxon tests (Demšar, 2006), corrected for multiple comparisons using the Bonferroni–Holm step‐down method.

## 3 RESULTS

**Figure 2:**
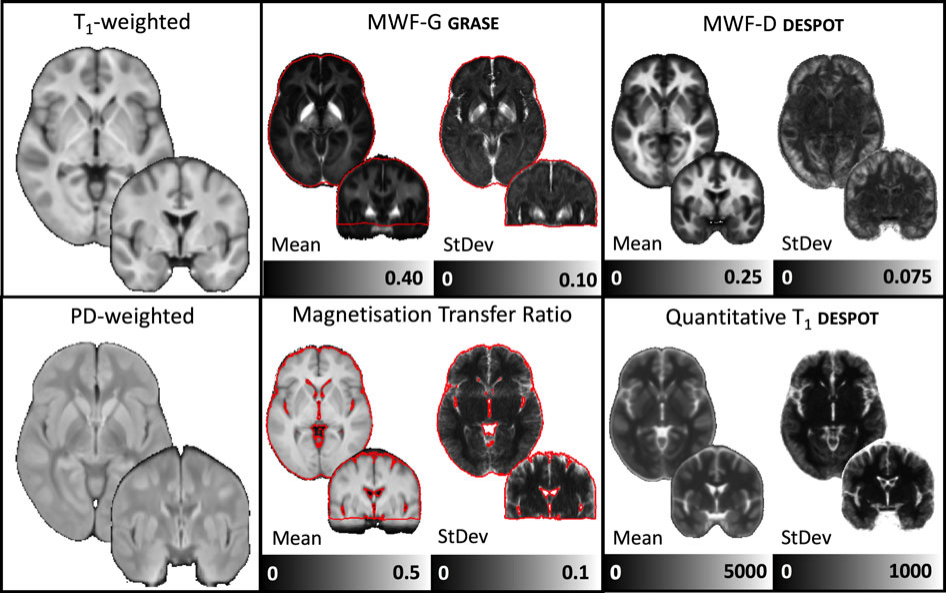
Population average images of the weighted and quantitative images for the healthy control cohort only. Red lines indicate the edge of coverage for all subjects in the myelin water fraction and magnetization transfer ratio images. Images are shown in MNI space on a coronal *Y* = −2 and an axial slice *Z* = −2 [Color figure can be viewed at http://wileyonlinelibrary.com]

Sample mean and *SD* maps of the 38 healthy control datasets are illustrated in Figure 2. In all four methods, the *SD* values are lower in WM than in gray matter, reflecting the fact that these are healthy adult controls (so no large deviations in WM content are expected). All analyses comparing methods or including the patient group were performed in standard MNI space and only in areas of the brain with coverage in all four modalities.

### 3.1 Correlations between techniques

**Figure 3:**
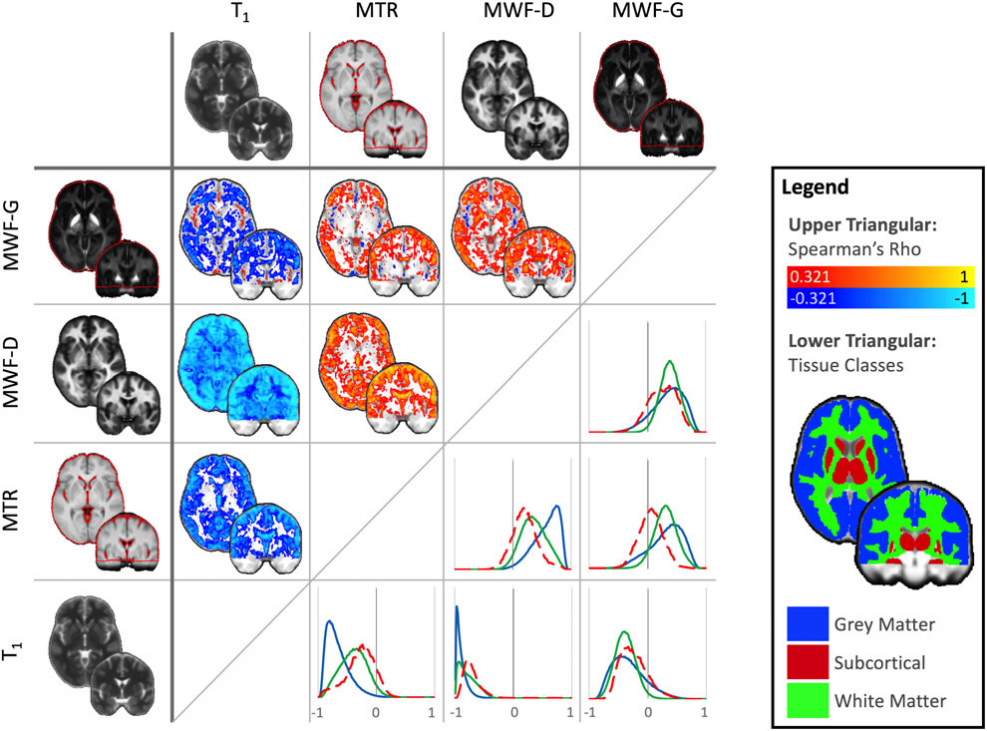
Cross‐sectional Spearman's rank correlations between quantitative techniques, calculated for every voxel (upper triangular) in the healthy control cohort. Normalized histograms of the voxelwise correlations are also plotted according to tissue class (lower triangular, correlation coefficient value bins on the *x*‐axis) with subcortical structures (red mask/dashed line), white matter (green mask/line), and gray matter (blue mask/line). Images are shown thresholded for visualization only at ρ > 0.321 or ρ < 0.321, *p* < 0.05 (two‐tailed, uncorrected). Raw unthresholded volumes are available as supplementary material. Images are shown in MNI space on a coronal *Y* = −2 and an axial slice *Z* = −2 [Color figure can be viewed at http://wileyonlinelibrary.com]

To assess the similarity between methods at capturing individual differences, voxelwise Spearman rank correlations between parameters are demonstrated in Figure 3 (upper triangular). The histogram of the correlation values for four different tissue classes in healthy controls are in the lower triangular. The correlation maps shown in the upper triangle of Figure 3 are also provided in nifti format on neurovault (https://neurovault.org/collections/4709/) and at https://www.msmri.com/.

Correlations across tissue classes (gray matter, WM, and subcortical gray structures) are reported in Table 3. These are reported separately for the healthy control population (*n* = 38) and the subset of patients for whom all four modalities were available (*n* = 24). The correlation coefficients (ρ) are summarized in Table 3. The most consistent correlations across all tissue classes were found between MWF‐D and qT_1_ (median ρ across tissue classes 0.80, mean ρ = 0.77) and MWF‐G and MWF‐D (median ρ = 0.59, mean ρ = 0.59). In lesional tissue, for the 24 patients for whom all quantitative modalities were available, qMRI estimates covaried between all pairs of methods.

**Table 3:** Pearson's correlation coefficients between average quantitative values across subjects in different tissue classes

								Patients (*N* = 24)									
Modality		Controls (*N* = 38)						Normal appearing						Pathological			
1	2		GM		WM		Subcortical		GM		WM	Subcortica			Lesion		Perilesion
MWF‐G	QT1		−0.24	*	−0.51		−0.09		−0.19	*	−0.71	*	−0.57	*	−0.66	*	−0.64
MWF‐G	MWF‐D	*	0.53	*	0.57	*	0.51		0.50	*	0.69	*	0.62	*	0.66	*	* 0.63
MWF‐G	MTR		0.23	*	0.54		−0.26		0.07		0.41		−0.10	*	0.57		0.48
MWF‐D	MTR	*	0.60		0.35		−0.11	*	0.54		0.25		0.17	*	0.54		0.37
MWF‐D	QT1	*	−0.71	*	−0.65	*	−0.56	*	−0.79	*	−0.81	*	−0.80	*	−0.97	*	−0.93
MTR	QT1	*	−0.90		−0.40		−0.14	*	−0.87		−0.44		−0.42	*	−0.54		−0.39

WM, white matter; GM, gray matter. Asterisk indicates *p* < 0.01

### 3.2 MS and healthy control comparisons

**Figure 4:**
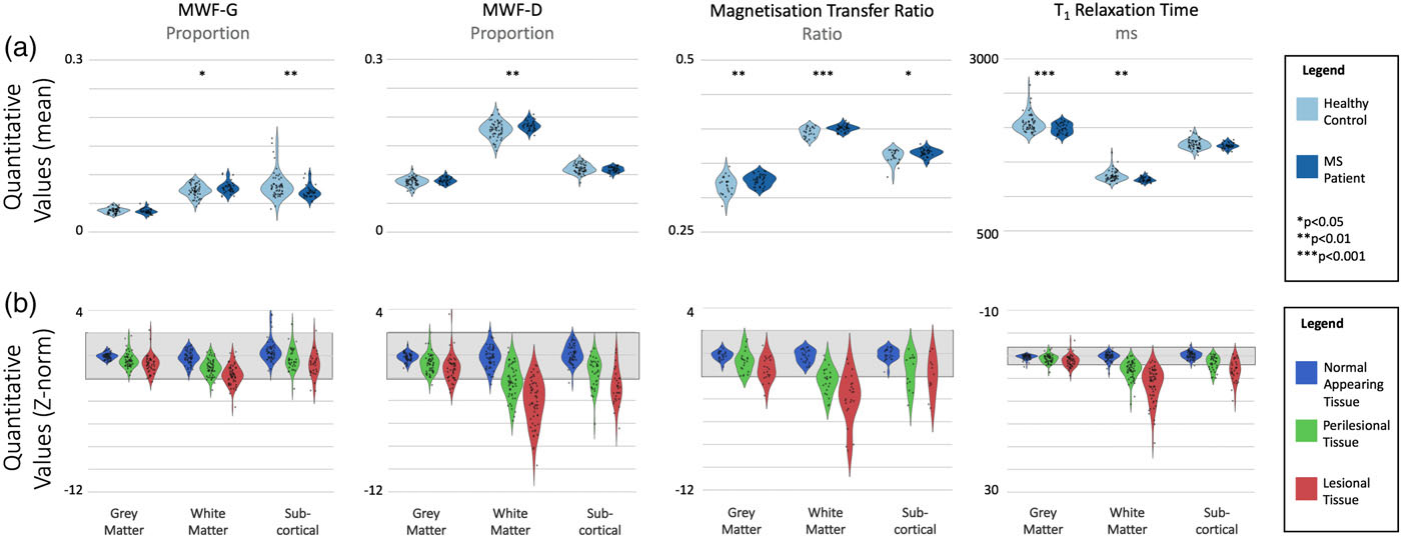
Top row (a): Average quantitative MRI values in three tissue classes for healthy controls and patients (normal appearing tissue only); and bottom row (b): The relative difference (Z scaled to the control sample, with control range indicated in gray) in individual patients in the three tissue classes according to whether the tissue is normal appearing, perilesional or lesional. In this part of the figure, the shaded area represents ±2 standard deviations of the healthy control population values. Note the larger scale on the *y*‐axis for T1 relaxation time [Color figure can be viewed at http://wileyonlinelibrary.com]

**Figure 5:**
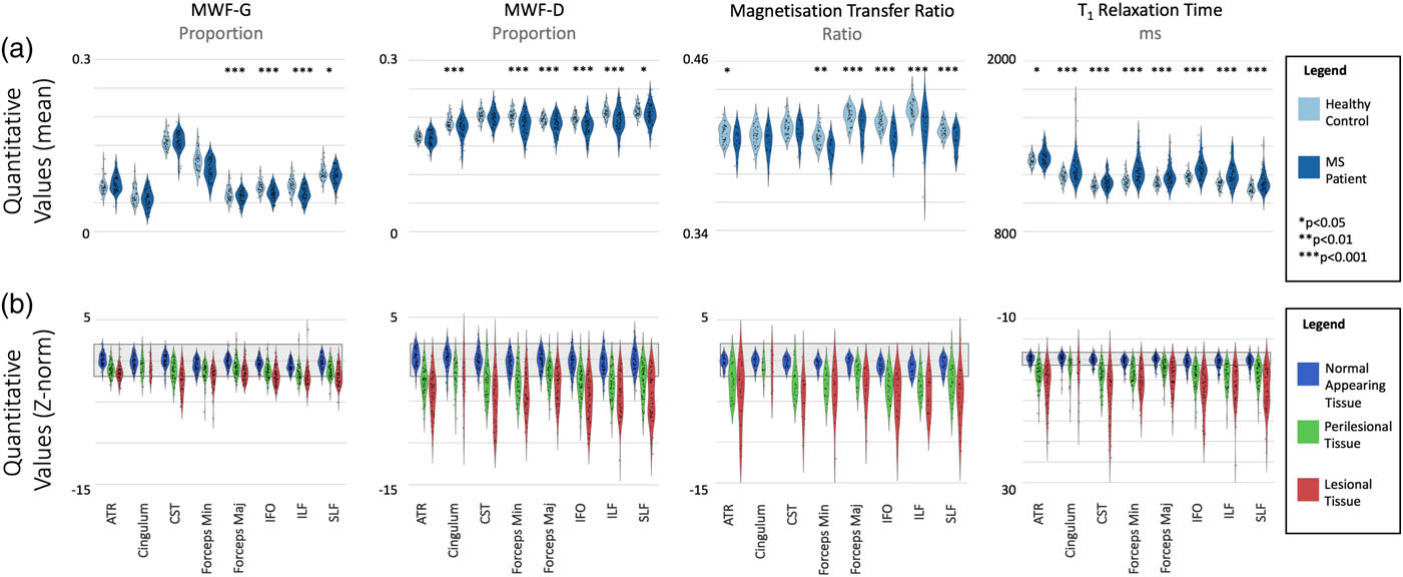
Top row (a): Average quantitative MRI values in eight white matter tracts (normal appearing tissue only); and bottom row (b): The relative difference (Z scaled to the control sample, with control range indicated in gray) in individual patients in the eight white matter tracts according to whether the tissue is normal appearing, perilesional or lesional. In this part of the figure, the shaded area represents ±2 standard deviations of the healthy control population values. ATR, anterior thalamic radiation; CST, corticospinal tract; IFO, inferior fronto‐occipital fasciculus; ILF, inferior longitudinal fasciculus; SLF, superior longitudinal fasciculus [Color figure can be viewed at http://wileyonlinelibrary.com]

Violin plots were created for each tissue class (gray matter, WM, and subcortical structures) in the patient and control groups (Figure 4a), indicating the spread of values for each region. In these analyses, all available data in the patient group was used (*n* = 24 for MTR, and *n* = 56 for the other methods). For all methods, significant differences between MS and controls were detectable between mean quantitative values in WM regions (*p* < 0.05, corrected for multiple comparisons). Notably, these summary values for each tissue type explicitly excluded tissue identified as lesional and peri‐lesional on clinical inspection. Figure 4b illustrates single subject Z‐scores for patients compared to the healthy control template. There was extensive overlap with the control average in the patient average for NAWM. Lesional and perilesional tissue had lower MWF‐D, MWF‐G, and MTR, and higher qT_1_ values, than the control average in lesional tissue for almost every individual patient. Similarly, Figure 5 demonstrates violin plots broken down into eight WM tracts. For both Figures 4 and 5, for healthy controls *N* = 38 for all metrics, while for MS patients *N* = 56 for MWF‐G, MWF‐D, and qT1; and *N* = 24 for MTR (see Table 1 for subject demographics).

### 3.3 AUC analysis

**Figure 6:**
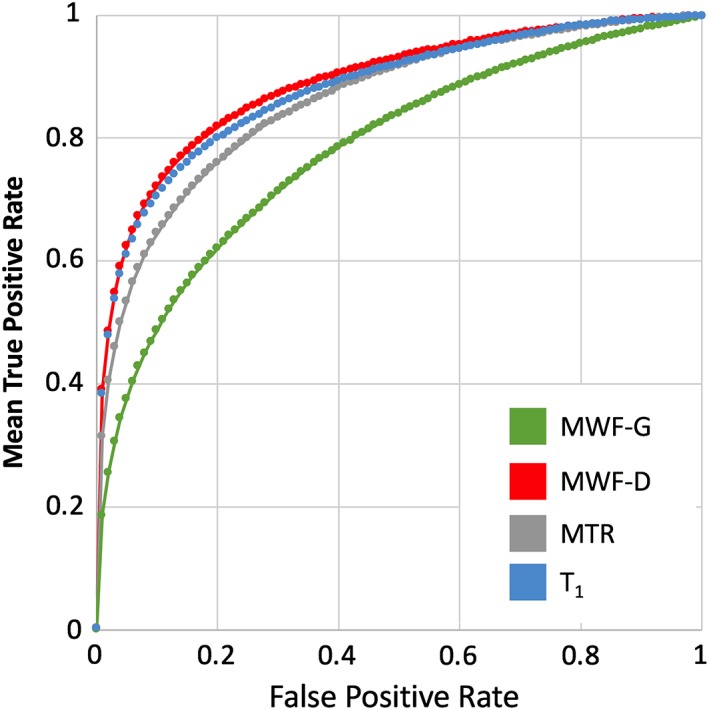
Receiver operator characteristic (ROC) curves for each quantitative MRI method showing accuracy of detecting manually labeled lesions by measuring voxel‐level difference from controls (Z‐score). Curves represent average true positive rates for fixed false positive rate values across the sub‐sample of 24 patients with MS (only patients with all 4 MRI [*n* = 24] modalities available were included in this analysis) [Color figure can be viewed at http://wileyonlinelibrary.com]

To quantify the relative sensitivity and specificity of the four quantitative MR techniques for detecting lesions, we calculated ROC curves for each individual patient. True positives were defined using the lesion masks, identified semi‐automatically with radiologist supervision. A nonparametric Friedman test indicated that the AUCs for detecting these delineated lesions were significantly different between the four quantitative MR techniques (*Χ* ^2^ = 32.350, df = 3, *p* < 0.001). Post hoc Wilcoxon signed rank tests (for paired data) indicated that MWF‐G had a significantly lower performance than all other methods (*p* < 0.001) and MWF‐D performed significantly better than qT_1_ (*p* = 0.017). MWF‐D had significantly higher AUC compared to both qT_1_ (*p* = 0.014) and MTR (*p* = 0.024). There was no significant difference between qT_1_ and MTR (*p* = 0.92). See Figure 6 for a plot of the ROC curves for each modality averaged over subjects. In this plot, each false discovery rate is fixed at the average (Fawcett, 2006). Importantly, the range of AUC values for MTR, qT_1_, and MWF‐G were only slightly (albeit significantly and consistently) different (see Figure 6 for average ROCs at fixed false positive rate thresholds). Table 4 has summary statistics of the AUC measures per modality.

**Table 4:** Summary of area under the curve (AUC) values for each technique

	Mean AUC (*SD*)		Range (min, max)	
MWF‐G	0.78	(0.12)	(0.46,	0.94)
MWF‐D	0.89	(0.08)	(0.65,	0.97)
MTR	0.86	(0.10)	(0.58,	0.96)
T_1_	0.88	(0.08)	(0.61,	0.97)

### 3.4 Sample quantitative imaging parameter summary maps and brain coverage

In place of estimating statistical power for a limited amount of study designs/effect sizes, we include group average mean and *SD* maps of each parameter for the healthy control group (Figure 2) and provided as files in nifti format on neurovault (https://neurovault.org/collections/4709/) and at https://www.msmri.com/. These summary statistics were calculated in MNI space.

## 4 DISCUSSION

In this work, we have quantified the in vivo relationship between four advanced quantitative MRI techniques each putatively related to myelin. Metrics calculated from each of these techniques (MWF‐G, MWF‐D, MTR, and qT1) showed strong relationships throughout the brain, though these relationships varied in different tissue types. The measures were all sensitive to differences in MS tissue compared to healthy controls, though to varying degrees, and had similar sensitivity profiles to manually identified MS lesions, with MWF‐D being the most accurate. The strong associations between MWF‐D and qT_1_ observed in the present study have been demonstrated previously by (De Santis et al., 2014), albeit using a region‐of‐interest based approach and looking at average values across subjects. Indeed, if any measure is specific to myelin, it should also show close correspondence to T_1_ in healthy tissue (Stüber et al., 2014).

However, in the MS brain specifically, the combination of increased water content, demyelination, and tissue iron deposition may disrupt this relationship. In the extreme case of completely demyelinated tissue, the inverse relationship between myelin and T_1_ would break down as the MWF maps will approach zero. Correlation coefficients between MWF‐D and MWF‐G indicated a moderate correlation in most tissue types. In fact, after MWF‐D and qT1, MWF‐D and MWF‐G were the pairs of measures significantly correlated across the most tissue classes. We observed weak, nonsignificant correlations in most tissue classes between MWF‐D and MTR. Similar to all other inter‐method comparisons, the correlation was significant in lesional tissue, indicating that although the methods may be measuring different quantities in healthy tissue, they are similarly sensitive in MS lesions.

There is a more established history in quantifying the similarity between MWF‐G, MTR, and qT_1_. Previous results have demonstrated a lack of correlation between MWF‐G and MTR in nonlesional WM (Vavasour et al., 2011). In an experimental autoimmune encephalomyelitis (EAE) guinea pig model, Gareau et al. (2000) suggested MTR was sensitive to inflammatory‐related changes to the structure of WM whereas MWF‐G was related to myelin content itself. They detected no relationship between MTR and MWF‐G. Here, we detected a positive relationship on average in healthy control WM and in lesional tissue in MS patients. In the MS patient group, NAWM did have a positive though nonsignificant relationship indicating here in patients (*n* = 24), and maybe the Gareau investigation (*n* = 24 as well), there may simply have been insufficient samples to detect weak relationships. Several in vivo studies suggest that MTR is most strongly influenced by water content (Fox et al., 2005; Giacomini et al., 2009; Vavasour et al., 2011; Van Waesberghe et al., 1997). In the present study, we find weak correlations between MTR and qT_1_ (which is strongly linked to water content) in WM, but similar to previous studies, we find a strong correlation in lesions, most likely due to an increase in water content.

In addition to characteristic focal lesions, there are diffuse abnormalities in the MS brain and notably: differences in brain volume (Stefano et al., 2010); increased edema (Filippi et al., 2012) and axonal swelling (Fisher et al., 2007); and diffuse changes in WM, as observed in the present study (Figures 4 and 5). We observed moderate global differences in the myelin‐related measures between controls and our MS cohort (Figure 4), similarly to previous studies (Kitzler et al., 2012; Faizy et al., 2016; Kolind et al., 2012; Oh, Han, Lee, Nelson, & Pelletier, 2007; Cercignani, Bozzali, Iannucci, Comi, & Filippi, 2001; Vavasour et al., 1998). When looking at smaller WM tracts, all MR measurements in the present study showed differences between subjects with MS and healthy controls in at least some regions (Figure 5). Comparing MS to controls, qT1 showed the highest number of significantly different regions (8), followed by MWF‐D and MTR (6), and then MWF‐G (4). These alterations in normal‐appearing tissue may reflect diffuse alterations in the WM. Multi‐component relaxation especially may be sensitive to subtle lesions that may be missed by radiological inspection (Laule et al., 2004). Additionally, cortical lesions may have downstream functional or structural effects on connected gray and WM tissue (Siffrin, Vogt, Radbruch, Nitsch, & Zipp, 2010). These nonlesional pathological features are likely represented in the performance curves of the modalities for lesion detection by apparently false positives (Figure 6). Cortical lesions are notoriously difficult to detect with both conventional and quantitative MRI (Calabrese & Castellaro, 2017), although with specific sequences, high field imaging, and ideally retrospective histopathological knowledge of lesion location, myelin content of cortical lesions can be investigated by mapping the lesions detected with these advanced techniques onto myelin‐sensitive MRI (Jonkman et al., 2016)] maps; unfortunately, none of these methods were available for this study.

One crucial aspect of individual differences relevant here is the effect of age on all of the quantities. Changes in WM tissue content occur throughout the lifespan (Callaghan et al., 2014) and, if investigating single patients, this change needs to be taken into account. In this study, we used an average and *SD* taken from a sample of healthy individuals with a wide age‐range, from 20 to 52 years. This is not ideal. MS can occur at any point throughout the lifespan and therefore detecting lesions or abnormalities needs to be performed in the context of the maturation of the brain at the time of the scan. What is still missing is a measure of each quantity appropriate to age, akin to a growth curve, though this has been developed in younger cohorts (Dean III et al., 2014; Sadeghi et al., 2013).

All quantitative MR measurements showed differences between lesions and NAWM. Compared to radiologist‐detected lesions, MWF‐D had the best performance in detecting these lesions (Figure 6), followed by qT1 and MTR. Good performance by qT1, in particular, is predictable since the presence of lesions on PD/T_2_‐weighted images is due to their increase in water content, and qT_1_ is greatly influenced by water content. Lesions identified by MWF‐G were the least similar to radiologist‐detected lesions. This could be because MWF‐G is only moderately correlated with water content (*r* = −0.36; Vavasour et al., 2011), and it is known that not all lesions show demyelination (Van Der Valk & De Groot, 2000).

ROC curves are used here as an indicator of *relative* sensitivity and specificity of the MRI methods, but their values can be misleading when the true positive/true negative ratio is very skewed, as is the case here. Lesion volumes were, on average, about 0.5% of the total WM volume mask used in this analysis. With these proportions, a 1% false positive rate could have twice as many false positive voxels as there are lesional voxels, even with perfect sensitivity (100 true positive rates). To improve on this, many studies have combined multiple image modalities, both qualitative and quantitative, to produce more accurate lesion segmentations (Brosch et al., 2016; Lladó et al., 2012; Mah, Jager, Kennard, Husain, & Nachev, 2014). Notably, combined analysis of MWF‐G and MTR over time has shown promise in distinguishing tissue injury and remyelination (McCreary et al., 2009).

ROC curves also miss another important factor in profiling tissue biomarkers in MS. Changes in both myelin and water content are known to occur (Laule et al., 2004), reflecting different aspects of the pathological processes. To accurately separate these features, any given technique should preferably be sensitive to one or the other, to distinguish which process is taking place. From this point of view, it is apparent here that qT_1_ is very sensitive to tissue changes (e.g., larger z scores than any other method in Figures 4 and 5) but not specific to which change is occurring in the tissue, for example, change in water content or myelin, reflected in the lower area under the curve in Figure 6 compared to MWF‐D. With the advantages and disadvantages of each technique, it is also necessary to consider the relative performance of each technique with regards to the acquisition time. The voxel volume for all sequences was ~5 mm^3^, with isotropic voxels and whole‐head coverage for mcDESPOT, whereas GRASE and MTR data were collected with 5 mm thick slices and limited coverage. With respect to time, GRASE has a disadvantage compared to mcDESPOT and MTR. GRASE is fundamentally limited by the need to acquire a sufficient number of data points in the T_2_‐decay, here 32 points going out to 320 ms, and a sufficiently long TR, in this study TR = 1,000 ms, to avoid T_1_‐weighting confounds, although recent developments may allow faster GRASE acquisition (Chen, Majumdar, & Kozlowski, 2014; Zhang et al., 2015). Sensitivity to tissue‐specific changes as well as acquisition time should be taken into account when selecting the appropriate quantitative technique to use in a study.

This brings up another methodological limitation when comparing the different methods. Limitations on matching image resolution across acquisitions mean that voxels can have different aspects of partial volume to each other. Therefore, while MWF‐D and qT_1_ are matched on voxel dimensions and are acquired in the same space, MWF‐G and MTR are highly anisotropic in the slice dimension. This is a risk, especially when investigating the association's voxelwise. In areas such as the ventricles (where there is strong partial volume in the inferior–superior axis), this could falsely induce or occlude associations between methods. Although spatial smoothing may help, matching acquisition dimensions can better address the problem. In addition, the fact that the mcDESPOT acquisition was used for nonlinear registration means that errors in registration are less likely for qT_1_ and MWF‐D compared to the other two methods where an additional rigid registration is needed and error may be introduced just to MTR and MWF‐G due to the compound registration approach. In all cases however, multiple interpolation steps were avoided by combining the calculated transformations and interpolating from native to MNI space in a single step.

In summary, we demonstrate that individual differences of WM tissue content are not consistent between WM quantification techniques in the same individuals. Although important in itself, this is especially important as these techniques are being used more and more in research, referred to interchangeably, though they are obviously quite different. These techniques are also being increasingly used in clinical trials of WM disorders and, where pathological tissue is concerned, we show reassuringly good correspondence between the techniques. However, depending on the exact purpose of a study, one technique may be preferred over another. For studies that require the greatest confidence in the interpretation of results as changes specific to myelin, MWF‐G has undergone the greatest degree of validation and is thought to provide the greatest specificity. If sensitivity to differences in tissue types (e.g., lesion vs. NAWM, NAWM vs. healthy control WM) is more important than the strictest specificity, MWF‐D and qT1 showed the greatest separation. Consequently, these techniques are the most likely to be sensitive to change over time. If acquisition or analysis times are severely limited, MTR or qT1 provide the most practical tools. To enable bespoke power analyses for future trials and studies, we provide voxelwise estimates of the sample mean and *SD* of the four quantitative MRI techniques (https://neurovault.org/collections/4709/).
